# Osmoreceptors do not exhibit a sex‐dependent modulation of forearm skin blood flow and sweating

**DOI:** 10.1002/phy2.226

**Published:** 2014-02-07

**Authors:** Juliana Barrera‐Ramirez, Ryan McGinn, Michael R. Carter, Hernan Franco‐Lopez, Glen P. Kenny

**Affiliations:** 1 Human and Environmental Physiology Research Unit, School of Human Kinetics, University of Ottawa, Ottawa, Ontario, Canada

**Keywords:** Osmolality, passive heating, sex‐differences, sweating

## Abstract

Studies show that increases in plasma osmolality result in a delayed onset threshold of thermoeffector responses. However, it remains unclear if there are sex‐related differences in the osmotically induced changes in both sweating and cutaneous vascular conductance (CVC). Nine young men and nine young women were passively heated (water‐perfused suit) to 1.5°C above baseline esophageal temperature while in an isosmotic (0.9% NaCl saline infusion) (ISO) and hyperosmotic (3% NaCl saline infusion) (HYP) state. Forearm sweat rate (ventilated capsule), skin blood flow (laser‐Doppler), esophageal temperature and skin temperature were continuously recorded. Sweat gland output (SGO) on the forearm was calculated from the number of heat activated sweat glands (modified iodine‐paper technique) at the end of heating. The onset threshold and thermosensitivity of sweating and CVC were determined using the linear portion of each response plotted against mean body temperature and analyzed using segmented regression analysis. We show that the osmotically induced delay in the onset threshold of sweating and CVC is similar between males and females. Although the thermosensitivity of CVC was similar between sexes (*P* = 0.601), the thermosensitivity of sweating was consistently lower in females compared to males (*P* = 0.018). The lower thermosensitivity in sudomotor response of females was accompanied by a lower SGO (*P* = 0.003), albeit similar sweat gland activation to males (*P* = 0.644). We conclude that sex‐related differences in thermoeffector activity are independent of osmoreceptor activation. Therefore, osmoreceptors do not exhibit sex‐related differences in the modulation of CVC and sweating responses during heat stress.

## Abstract (precis)

To assess if there are sex‐related differences in the osmotically induced changes in both sweating and cutaneous vascular conductance (CVC), nine young men and nine women were passively heated while in an isosmotic (0.9% NaCl saline infusion, ISO) and hyperosmotic (3% NaCl saline infusion, HYP) state. No sex‐related difference in the osmotically induced delay in the onset threshold for sweating and CVC was measured. Although the thermosensitivity of CVC was similar between sexes under both the ISO and HYP condition, the thermosensitivity of sweating was consistently lower in females compared to males. We show that the sex‐related differences in thermoeffector activity are independent of osmoreceptor activation.

## Introduction

While thermoeffector responses (sweating and skin blood flow) are primarily modulated by changes in internal and skin temperature (i.e., thermal factors), these responses can also be influenced by factors of nonthermal origin (i.e., baroreceptors, mechanoreceptors, metaboreceptors, central command, and osmoreceptors) (Shibasaki et al. 2003; Kenny and Journeay 2010). Furthermore, this nonthermal sensory receptor modulation of heat loss responses may differ between males and females. For instance, response to baroreceptor activation has been shown to differ between males and females such that females exhibit lower baroreflex buffering of blood pressure (Convertino 1998; Christou et al. 2005) and lower orthostatic tolerance (Shoemaker et al. 2001). It has been further suggested that the nonthermal baroreceptor‐mediated attenuation of thermoefferent responses may be more pronounced in females than males, as exemplified by higher onset thresholds of sweating and cutaneous vascular conductance (CVC) in females post exercise (Kenny et al. 2007). Similarly, sex‐related differences have been observed in both metabo‐ (Jarvis et al. 2011) and mechanoreflexes (Ives et al. 2013), with females exhibiting attenuated responses when compared to males. Even though extensive research has been devoted to the understanding of sex‐related differences in nonthermal factors, it remains unclear whether osmoreceptors also exhibit a sex‐specific response.

Given that prolonged exercise and/or exposure to heat can result in profound fluid loss through sweating (and thereby increases in osmolality) (Sawka et al. 2001), examination of sex‐related differences in the osmoreceptor modulation of thermoeffector responses remains an important area of consideration. Numerous studies have demonstrated that hyperosmolality impairs heat loss responses as evidenced by a delayed onset threshold of both sweating and skin blood flow (Fortney et al. 1984; Takamata et al. 1995a, 1997; Shibasaki et al. 2009; Lynn et al. 2012), with no effect on the thermosensitivity of either response (Takamata et al. 1997; Lynn et al. 2012). However, it is still unknown whether this impairment varies between males and females. Recent studies on fluid regulation have noted that there are sex‐differences in the osmotically driven release of vasopressin, with females having reduced vasopressin concentrations than males for a given level of plasma osmolality (Stachenfeld et al. 2001). This suggests that for a given absolute value of osmolality, there is a different response between males and females. Therefore, it is plausible to suggest that sex‐related differences in the osmoreceptor modulation of heat loss responses may also exist.

Thus, the purpose of this study was to examine if there are sex‐related differences in the osmotically induced changes in both sweating and CVC during whole‐body heat stress. We hypothesized that (1) an increase in osmolality will result in a delayed onset threshold of both sweating and CVC in males and females; and (2) the overall delay in onset threshold will be higher in females than in males. This will be accomplished using whole‐body passive heating to increase core temperature and promote increases in sweating and skin blood flow.

## Methods

### Ethical approval

The present study was approved by the University of Ottawa Health Sciences and Science Research Ethics Board in accordance with the *Declaration of Helsinki*. Written informed consent was obtained from all volunteers prior to participating in the study.

### Participants

A total of nine young men (25 ± 5 years) and nine young women (22 ± 4 years) were recruited for this study. Participants were healthy, nonsmokers and had no cardiovascular, renal, respiratory, or metabolic conditions. None were taking any prescription or over‐the‐counter medication, with the exception of oral contraceptives in some female participants (*n* = 4). Female participants completed the experimental sessions during the follicular phase of the menstrual cycle or the placebo/no‐pill week of oral contraceptive use. Participant characteristics are presented in Table 1.

**Table 1.** Participant characteristics.

Sex	Age (years)	Body mass (kg)	Height (cm)	Baecke index	Kohl (MET‐h/week)
Males	25 ± 5	76.16 ± 6.51^*^	175 ± 8^*^	2.99 ± 0.56	25.71 ± 10.0
Females	22 ± 4	60.44 ± 8.38	166 ± 5	2.92 ± 0.76	24.87 ± 15.2

* Significantly different from females (*P* ≤ 0.05). Values represent mean ± standard deviation.

### Experimental protocol

#### Preliminary session

Participants completed a Physical Activity Readiness Questionnaire (Par‐Q) and an American Heart Association/American College of Sports Medicine Health/Fitness Facility Preparticipation Screening Questionnaire to assess their eligibility to participate in the study. In order to determine levels of physical activity, participants completed the Baecke Sport Index Questionnaire and the Kohl Physical Activity Questionnaire (MET‐h/week).

#### Experimental sessions

This study consisted of two experimental sessions. Female participants completed the experimental trials during the first 8–10 days after the start of their self‐reported menses (follicular/low hormone phase). Females taking oral contraceptives completed the trials during the placebo/no‐pill week. Hormone (17*β*‐estradiol and progesterone) status was confirmed on the day of each trial.

Participants reported to the laboratory between 8:00 am and 11:00 am on each experimental session. They were asked to refrain from salty food, caffeinated beverages, alcohol, and exercise 24 h prior to the test day. In addition, participants were asked to hydrate well before the trial. Hydration status was verified upon arrival to the laboratory by providing a urine sample and measuring urine specific gravity.

Upon arrival to the laboratory, participants changed into shorts and a sports bra (females) and provided a urine sample after which a nude weight was obtained. Participants then donned a liquid‐perfusion garment (Allen‐Vanguard Corp., Ottawa, ON, Canada) which covered the entire body except for the head, hands, feet, and right forearm. They then rested in supine posture for a baseline period of 30 min to ensure the stabilization of fluid compartments in the body before obtaining a baseline blood sample. During the baseline rest, a venous catheter was inserted in the right arm and connected to a secondary line in order to conduct a 90‐min infusion of either isosmotic 0.9% NaCl (ISO) or hyperosmotic 3% NaCl (HYP) saline to maintain or increase blood osmolality, respectively. The saline solutions were infused at a rate of 0.2 and 0.125 mL·min^−1^·kg^−1^ of body weight for 0.9% and 3.0% NaCl, respectively. These infusion rates have successfully been used in previous studies (Takamata et al. 1997; Lynn et al. 2012). Throughout the infusion period, blood pressure and heart rate were measured continuously, while thirst sensation ratings were obtained every 10 min. Baseline cardiac output measurements were obtained in duplicate during the last 10 min of the infusion period. Furthermore, the suit worn by the participants was perfused with water at 34°C to stabilize skin temperature. In addition, two sweat capsules and two laser‐Doppler probes along with the heater modules (maintained at 35°C) were placed in the right forearm. At the end of the infusion a second blood draw was collected to confirm osmotic state in each session.

Upon completion of the saline infusion, the water being perfused through the suit was heated to 48.5°C and participants were covered with a thick blanket to minimize any heat lost to the environment. Whole‐body passive heating continued until esophageal temperature (*T*_es_) had increased by 1.5°C from baseline (i.e., end of infusion). Throughout the heating protocol, blood pressure and heart rate were measured continuously. Thirst sensation ratings were recorded every 0.25°C increase in *T*_es_. Cardiac output and blood samples were obtained every 0.5°C increase (i.e., at 0.5°C, 1.0°C, and 1.5°C) in *T*_es_. Upon completing the heating protocol (i.e., 1.5°C above baseline *T*_es_), a sweat gland activation assessment was performed followed by a maximal skin blood flow measurement consisting of a 20‐min local heating period at 42°C and a subsequent 25‐min period at 44°C. Before leaving the laboratory, participants provided a second nude weight and a urine sample.

### Measurements

Esophageal temperature was measured using a pediatric thermocouple probe of 2 mm in diameter (Mon‐aTherm Nasopharyngeal Temperature Probe, Mallinckrodt Medical, St‐ Louis, MO). The probe was inserted through the participant's nostril and down the esophagus to the length of the probe (~40 cm) with the end of the probe sitting at approximately heart level for most individuals of average height. The probe was inserted while participants swallowed water through a straw. Skin temperature was measured at 10 points using T‐type (copper/constantan) thermocouples integrated into heat flow sensors (Concept Engineering, Old Saybrook, CT). The thermocouples were placed in the forehead, bicep, forearm, chest, abdomen, upper back, lower back, quadriceps, hamstrings, and front calf. All of these areas were previously shaved and thoroughly cleaned with alcohol. Each thermocouple was secured with a double‐sided adhesive ring and surgical tape (Blenderm, 3M, St. Paul, MN). Mean skin temperature was calculated using a 10‐point weighting of the regional proportions determined by Hardy and DuBois (1938). Temperature data were collected with an HP Agilent data acquisition module (model 3497A; Agilent Technologies Canada Inc., Mississauga, Canada) at a rate of one sample every 15 sec and simultaneously recorded on a personal computer with LabVIEW software (LabVIEW 7.0; National Instruments, Austin, TX).

Blood pressure was measured using a Finometer (Finapress Medical Systems, Amsterdam, Netherlands). Mean arterial pressure (MAP) was estimated from the beat‐to‐beat recording of the left middle finger arterial pressure waveform with the volume‐clamp method (Penaz 1973). Prior to measurement recording, the Finometer was calibrated using the physiocal criteria followed by brachial artery pressure reconstruction (Gizdulich et al. 1996, 1997). Heart rate was recorded continuously using a Polar‐coded transmitter and advantage interface (Polar Electro Oy, Kempele, Finland). Cardiac output (L·min^−1^) was measured noninvasively using an Innocor™ inert gas rebreathing unit with breath‐by‐breath ergospirometry and an arterial oxygen saturation sensor (Innovisions, Odense, Denmark). This system has been previously validated against the direct Fick method and thermodilution (Peyton and Thompson 2004). The following mixture of gases was employed: 5% nitrous oxide and 1% sulfur hexafluoride diluted with ambient air (Ayotte et al. 1970). The participant was asked to breathe through a 3‐way valve connected to a breathing filter (Pro‐Tec Filters, PF30S, 30 mm ports, Odense, Denmark), an antistatic rubber bag, and a gas analyzer. Breathing frequency was fixed at 20 breaths·min^−1^ with the assistance of an auditory metronome. Before each measurement, the rubber bag was filled with the mixture of gas with a volume previously determined for each participant, which enabled the participant to fully empty the bag with each inhalation. Stroke volume was calculated as cardiac output divided by heart rate.

Local sudomotor activity was measured on the right forearm in duplicate using 3.8 cm^2^ ventilated plastic capsules. The right forearm was shaved and cleaned with alcohol prior to placing and securing the sweat capsules with double‐sided adhesive rings, topical skin glue (Collodion HV; Mavidon Medical products, Lake Worth, FL), and surgical tape. Each capsule was ventilated with anhydrous compressed air at a rate of 1 L·min^−1^. Water content of the effluent air was measured using dew point mirrors (RH systems, Albuquerque, NM). Local sweat rate was calculated using the difference in water content between effluent and influent air and multiplied by the flow rate, and normalized for the skin surface area under the capsule (expressed in mg·min^−1^·cm^−2^). The number of heat activated sweat glands in the forearm was measured in triplicate in an area adjacent to the sweat capsules using the modified iodine‐paper technique (Gagnon et al. 2012) at the end of the passive heating protocol (i.e., at 1.5°C above baseline *T*_es_). Sweat gland output was calculated by dividing the corresponding sweat rate by the number of activated sweat glands.

Local skin blood flow was assessed in duplicate using laser‐Doppler velocimetry (PeriFlux System 5000; Perimed AB, Stockholm, Sweden) at the right forearm. Each laser‐Doppler flow probe was placed adjacent to the sweat capsules and secured with double‐sided adhesive rings in an area of the forearm that was not highly vascularized as determined by visual inspection. The laser‐Doppler flow probes remained in the same place for the duration of the experimental session to ensure reliable measurements. CVC was calculated as skin blood flow perfusion units divided by MAP and expressed as a percentage of maximum.

Plasma osmolality, serum osmolality, and plasma volume changes were determined using venous blood samples. An indwelling venous catheter was inserted in the antecubital vein of the right arm connected to a Luer‐Lock extension (Microbore Extension, Clave™ [Hospira Inc., Lake Forest, IL], Locking Spin Collar, Non‐DEHP) and secured in place with a 6 × 7 cm film dressing (Tegaderm Film, 3M Health Care, St. Paul, MN). Venous blood (~10 mL) was collected without stasis into K2 EDTA™, Serum™, and Lithium‐Heparin™ vacutainers (BD Vacutainer, Franklin Lakes, NJ) for hematology, serum, and plasma osmolality analysis, respectively. Blood samples in the K2 EDTA™ vacutainer were immediately analyzed for hemoglobin (Hb) concentration and hematocrit (Hct) ratio. Blood samples in the Lithium‐Heparin™ vacutainer were immediately centrifuged for 10 min and the aliquot was transferred to a plastic collection vile. Blood samples in the Serum™ vacutainer were allowed to sit for 20 min to allow the blood to fully coagulate before being centrifuged for 10 min and the aliquot separated. Both plasma and serum aliquots were immediately analyzed upon separation to determine osmolality using the freezing‐point method (Osmometer; Advance Instruments, Norwood, MA). An additional sample of blood was collected without stasis into a SST™ (BD Vacutainer, Franklin Lakes, NJ) vacutainer from female participants in order to confirm that each experimental session took place during the follicular phase of the menstrual cycle or placebo/no‐pill week of oral contraceptives. Samples were sent to an external laboratory (Gamma‐Dynacare Medical Laboratories, Ottawa, ON, Canada) for the analysis of 17*β*‐estradiol and progesterone.

Nude weight was recorded before and after each session using a calibrated scale (Mettler Toledo [Columbus, OH], Model KCC, USA). Urine specific gravity was determined in duplicate using a handheld refractometer (TS400, Reichter Inc., Depew, NY) before and after each experimental session. Thirst sensation was assessed using the Visual Analogue Thirst Sensation Scale (Marks et al. 1988), which has been shown to provide a high correlation between thirst and plasma osmolality during hypertonic infusions (Takamata et al. 1995b; Stachenfeld et al. 1996). Participants were presented with a scale of 175 mm in length where the lower end was labeled as “not thirsty at all”, at the length of 125 mm a horizontal line was labeled as “extremely thirsty” and a final line at 175 mm marked the end of the scale. Participants were instructed to draw a line at any point on the scale as they deemed appropriate. Thirst sensation was recorded as the distance (in cm) from the lower end to the mark drawn by the participant. To eliminate bias in the assessment of thirst sensation, this study followed a single‐blind design. Although all participants were informed about the type of saline infusions used in the study, the type of saline being used in each experimental session was not disclosed.

### Data analysis

Throughout the experimental protocol, local sweat rate and skin blood flow were measured in duplicate and the averages of both sites were used for statistical analysis. Mean body temperature was calculated using the following equation (Gisolfi and Wenger 1984; Wingo et al. 2010):
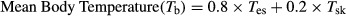
where *T*_es_ is esophageal temperature and *T*_sk_ is mean skin temperature. This equation was selected because it accounts for the well‐established influence of internal and skin temperature on heat loss (Nadel et al. 1971; Wenger et al. 1985). The onset threshold and thermosensitivity of sweating and CVC were determined using the linear portion of each response plotted against mean body temperature and analyzed using segmented regression analysis (GraphPad Prism 5.0; GraphPad Software, La Jolla, CA) (Cheuvront et al. 2009). Each measurement was also verified by visual inspection. The onset threshold was defined as the mean body temperature at which sweating and CVC rapidly increased. Similarly, thermosensitivity was defined as the slope of the linear portion of the thermoeffector response–mean body temperature relationship. Changes in plasma volume from baseline resting were calculated from changes in hemoglobin and Hct using the following equation (Dill and Costill 1974):
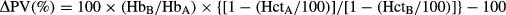
where ΔPV is the percent change in plasma volume, Hb_B_ is the hemoglobin concentration at baseline, Hb_A_ is the hemoglobin concentration at a specific time point (i.e., end infusion, 0.5°C, 1.0°C, 1.5°C), Hct_A_ is the hematocrit at a specific time point (i.e., end infusion, 0.5°C, 1.0°C, 1.5°C), and Hct_B_ is the hematocrit at baseline. Finally, thirst sensation ratings were calculated from the Visual Analogue Scale by subtracting the distance from the start of the scale to the line drawn by the participant from the total length of the scale (175 mm), this was divided by the total length of the scale and then multiplied by 100 to express it as a percentage.

### Statistical analysis

A two‐by‐two mixed analysis of variance (ANOVA) with the repeating factor of condition (levels: ISO and HYP) and the nonrepeating factor of sex (levels: males and females) was used to determine the effect of condition and sex on the onset threshold and thermosensitivity of both sweating and CVC, as well as on measures of urine specific gravity. A three‐way mixed ANOVA with the repeating factors of condition (levels: ISO and HYP), time (levels: pre, end infusion, at 0.5°C, at 1.0°C and at 1.5°C), and the nonrepeating factor of sex (level: males and females) was used to analyze the following variables: osmolality, plasma volume, MAP, cardiac output, heart rate, stroke volume, and thirst sensation. Holm–Bonferroni post hoc analysis of preplanned comparisons was carried out whenever an ANOVA was rejected. Independent samples *t*‐tests were performed to compare participants’ characteristics, Baecke Sport Index, and Kohl Physical Activity MET‐h/week between males and females. Similarly, paired samples *t*‐tests were employed to compare 17ß‐estradiol and progesterone levels in females during the ISO and HYP conditions. The level of significance was set to an alpha level of *P* ≤ 0.05. All statistical analysis was performed using SPSS 20.0 software package for Windows (SPSS Inc., Chicago, IL).

## Results

Male participants were taller (*P* = 0.010) and heavier (*P* < 0.001) than their female counterparts. There were no statistically significant differences in training status between males and females as estimated by the Baecke Sport Index Questionnaire (*P* = 0.821) or the Kohl Physical Activity Questionnaire (MET‐h/week) (*P* = 0.891) (Table 1). Female participants completed both experimental sessions during the follicular phase/no‐pill week of oral contraceptives. 17*β*–estradiol levels during the ISO and HYP were 143 ± 74 and 156 ± 92 pmol/L (*P* = 0.604), respectively. Similarly, progesterone levels for ISO and HYP conditions were 1.7 ± 0.5 and 1.8 ±0.4 nmol/L (*P* = 0.393), respectively. Both 17*β*–estradiol and progesterone levels were within the accepted reference range of 46–607 pmol/L and 0.6–4.7 nmol/L, respectively.

### Hydration status

No differences were observed in hydration status as determined by nude weight measurements between conditions in either males (ISO: 76.33 ± 6.56 kg; HYP: 76.52 ± 6.46 kg) or females (ISO: 60.19 ± 7.89 kg; HYP: 60.17 ± 8.53 kg) (*P* = 0.747). Furthermore, urine specific gravity measurements were also similar between conditions (*P* = 0.203) in both males (ISO: 1.013 ± 0.006; HYP: 1.017 ± 0.006) and females (ISO: 1.017 ± 0.004; HYP: 1.017 ± 0.006), as well as between sexes (*P* = 0.419).

### Blood osmolality and changes in plasma volume

**Figure 1.**
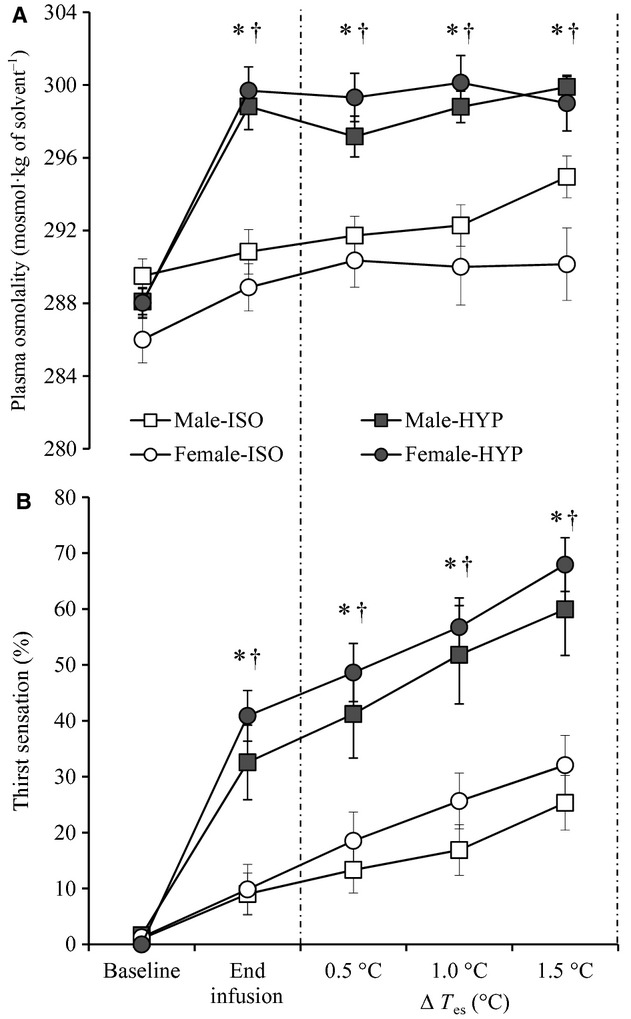
Plasma osmolality (A) and thirst sensation ratings (B) for males (□) and females (○) during isosmotic (ISO) and hyperosmotic (HYP) conditions throughout the experimental protocol (i.e., baseline, end of infusion and throughout passive heating). Values represent mean ± standard error. *Male‐HYP is significantly different from Male‐ISO and ^†^Female‐HYP is significantly different from Female‐ISO (*P* ≤ 0.05).

Baseline plasma osmolality measured before the start of saline infusion in both males and females were ISO: 290 ± 3 versus 287 ± 3 mosmol·kg of solvent^−1^ and HYP: 288 ± 2 versus 288 ± 2 mosmol·kg of solvent^−1^. A main effect of condition (ISO vs. HYP) in changes of plasma osmolality was observed (*P* < 0.001), while no main effect of sex was measured (*P* = 0.388). Infusion of hypertonic saline resulted in an increase in plasma osmolality from baseline levels (males: 299 ± 4 and females: 300 ± 4 mosmol·kg of solvent^−1^) (all *P* < 0.001), while infusion of isotonic saline resulted in a similar plasma osmolality at the end of infusion compared to baseline resting values (males: 291 ± 4 and females: 289 ± 4mosmol·kg of solvent^−1^) (all *P* > 0.06) (Fig. 1A). The increase in plasma osmolality observed during the HYP condition can be equivalent to 40 min of strenuous exercise or a 24‐h period of water deprivation (Bourque 2008). Therefore, this level of hyperosmolality represents a physiological relevant condition.

Similarly, baseline serum osmolality for males and females were ISO: 289 ± 2 versus 287 ± 4 mosmol·kg of solvent^−1^ and HYP: 289 ± 3 versus 289 ± 3 mosmol·kg of solvent^−1^. Similar to plasma, serum osmolality increased from baseline after infusion of hypertonic saline (males: 299 ± 3 and females: 300 ± 4 mosmol·kg of solvent^−1^) (all *P* < 0.001), while infusion of isotonic saline did not change osmolality levels (males: 290 ± 3 and females: 288 ± 3 mosmol·kg of solvent^−1^) (all *P* > 0.07). There was a main effect of condition (ISO vs. HYP) in changes of serum osmolality (*P* < 0.001), but no differences were observed between sexes (*P* = 0.277).

Changes in plasma volume at the end of infusion for males and females were ISO: +5.2 ± 3.4% versus +5.2 ± 4.0% and HYP: +7.6 ± 3.1% versus +8.1 ± 3.3%. These changes in plasma volume were not different between males and females (*P* = 0.668), however, there was a main effect of condition (i.e., ISO vs. HYP) within groups (*P* ≤ 0.001). Plasma volume decreased from end‐infusion levels with passive heat stress (*P* ≤ 0.001), although the decrease was similar between sexes (*P* = 0.153) and across conditions (*P* = 0.394).

### Thirst sensation

Infusion of hypertonic saline resulted in a significantly higher thirst sensation in comparison to isotonic saline (*P* < 0.001). This increased thirst sensation during HYP was similar between males and females (*P* = 0.392). Figure 1B shows the changes in thirst sensation over time.

### Thermal responses

#### Heating time

The total time required to increase *T*_es_ by 1.5°C from baseline for males and females were (ISO: 60 ± 11 vs. 56 ± 14 min; and HYP: 55 ± 8 vs. 51 ± 9 min). Heating time was significantly lower during the hypertonic condition compared to isotonic (*P* = 0.001), however, no differences between males and females were observed (*P* = 0.433).

#### Skin and core temperatures

Table 2 summarizes baseline and total change in esophageal temperature, mean skin temperature, and mean body temperature for both males and females during the ISO and HYP conditions. There were no differences in baseline esophageal temperature between conditions (*P* = 0.951), however, there was a main effect of sex (*P* = 0.009). Baseline mean skin temperature did not differ between conditions (*P* = 0.099) or sexes (*P* = 0.699). Furthermore, baseline mean body temperature was not different between conditions (*P* = 0.607), but it was statistically different between males and females (*P* = 0.026). Whole‐body passive heating resulted in a similar increase between males and females in esophageal (*P* = 0.505), mean skin (*P* = 0.326), and mean body temperatures (*P* = 0.933). Moreover, no differences were observed in the total change in esophageal (*P* = 0.543), mean skin (*P* = 0.869), and mean body temperatures (*P* = 0.745) between conditions (i.e., ISO and HYP).

**Table 2.** Baseline and total change in esophageal temperature (°C), mean skin temperature (°C), and mean body temperature (°C) for both males and females during the ISO and HYP conditions.

Condition	Sex	*T*_es_		*T*_sk_		*T*_b_	
		Baseline	∆*T*_es_	Baseline	∆*T*_sk_	Baseline	∆*T*_b_
ISO	Male	36.68 ± 0.19^*^	1.62 ± 0.13	33.80 ± 0.42	3.92 ± 0.50	36.10 ± 0.17^*^	2.08 ± 0.17
	Female	36.93 ± 0.38	1.56 ± 0.21	33.85 ± 0.57	4.13 ± 0.65	36.31 ± 0.38	2.08 ± 0.15
HYP	Male	36.63 ± 0.19^*^	1.64 ± 0.12	33.94 ± 0.51	3.85 ± 0.60	36.09 ± 0.19^*^	2.09 ± 0.20
	Female	36.97 ± 0.26	1.59 ± 0.26	34.07 ± 0.59	4.14 ± 0.76	36.39 ± 0.26	2.10 ± 0.21

*T*_es_, esophageal temperature; ∆*T*_es_, change in esophageal temperature; *T*_sk_, mean skin temperature; ∆*T*_sk_, change in mean skin temperature; *T*_b_, mean body temperature; ∆*T*_b_, change in mean body temperature; ISO, isotonic condition; HYP, hypertonic condition.

* Significantly different from females (*P* ≤ 0.05). Values represent mean ± standard deviation.

#### Sweating

**Figure 2.**
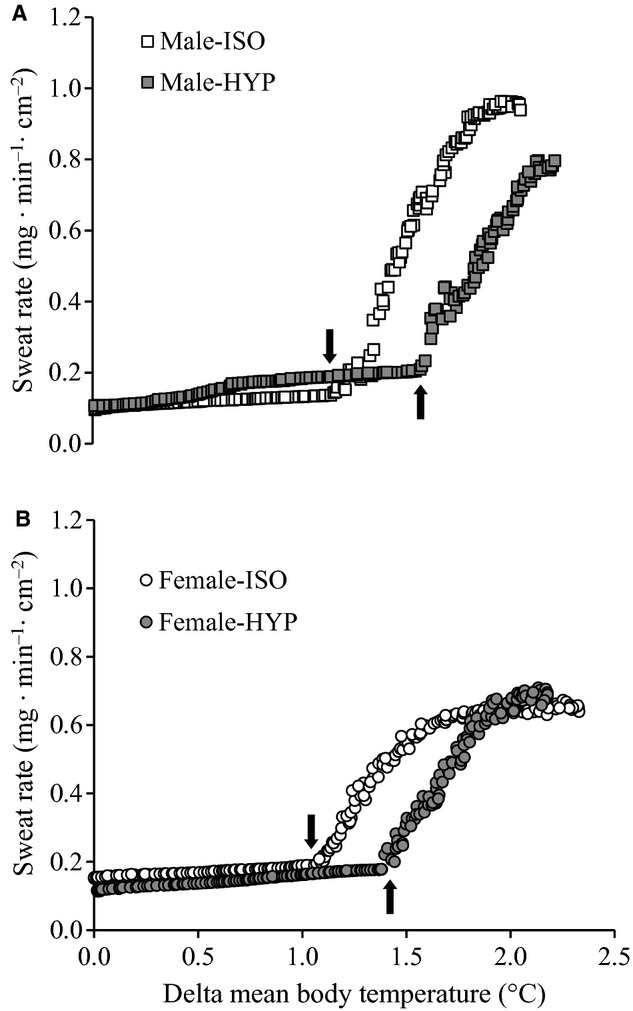
Forearm sweating response as a function of increases in mean body temperature during whole‐body passive heating in isotonic (open symbols) and hypertonic (closed symbols) conditions for two representative subjects (A: male; B: female). Arrows indicate the onset threshold for sweating.

Table 3 outlines the mean body temperature absolute onset threshold, change in onset threshold and thermosensitivity of local sudomotor response in both males and females. The absolute mean body temperature at which the onset of forearm local sweat rate occurred was higher in HYP compared to ISO (*P* < 0.001). Furthermore, a main effect of sex was observed in the absolute onset of sweating (*P* = 0.004), with females exhibiting a higher absolute onset. The change in mean body temperature from baseline to the onset of sweating was significantly higher during HYP (*P* < 0.001), however, there were no differences in the relative change in mean body temperature between males and females (*P* = 0.522) (Table 3). Finally, the thermosensitivity of sweating as represented by the slope of the sweating–mean body temperature relationship was similar between conditions (ISO vs. HYP) (*P* = 0.186). However, the thermosensitivity was consistently lower in females than in males (*P* = 0.018). The number of heat‐activated sweat glands per cm^2^ during the isotonic condition did not differ between sexes (*P* = 0.644) or condition (*P* = 0.678) (Table 4). Contrary to these results, total sweat gland output was consistently lower in females than males (*P* = 0.003) irrespective of condition (Table 4). The sweating response of two representative participants during ISO and HYP conditions is shown in Figure 2.

**Table 3.** Mean body temperature onset threshold and thermosensitivity of sweating and CVC in both males and females during ISO and HYP conditions.

Condition	Sex	Sweating			CVC		
		Threshold (°C)	Δ*T*_b_ (°C)	Slope (mg∙min^−1^∙cm^−2^/°C)	Threshold (°C)	Δ*T*_b_ (°C)	Slope (%CVC_max_/°C)
ISO	Male	37.12 ± 0.24^*†^	1.02 ± 0.30^†^	1.53 ± 0.70^*^	36.89 ± 0.32^*†^	0.78 ± 0.27^†^	83.26 ± 33.07
	Female	37.39 ± 0.26^†^	1.08 ± 0.24^†^	0.92 ± 0.26	37.25 ± 0.28^†^	0.93 ± 0.23^†^	97.13 ± 29.78
HYP	Male	37.38 ± 0.30^*^	1.30 ± 0.33	1.31 ± 0.57^*^	37.12 ± 0.29^*^	1.05 ± 0.29	86.64 ± 25.44
	Female	37.80 ± 0.18	1.41 ± 0.26	0.83 ± 0.34	37.58 ± 0.20	1.19 ± 0.34	85.21 ± 29.70

CVC, cutaneous vascular conductance; Δ*T*_b_, increase in mean body temperature; ISO, isosmotic condition; HYP, hyperosmotic condition.

*Significantly different from females and ^†^ significantly different from HYP (*P* ≤ 0.05). Values represent mean ± standard deviation.

**Table 4.** Total number of heat activated sweat glands and sweat gland output per gland in both males and females during ISO and HYP conditions.

Condition	Sex	HASG (number per cm^2^)	SGO (*μ*g/gland)
ISO	Male	95 ± 32	10.97 ± 4.23^*^
	Female	107 ± 28	6.11 ± 2.08
HYP	Male	100 ± 30	9.04 ± 2.88^*^
	Female	99 ± 18	5.42 ± 1.29

HASG, heat activated sweat glands; SGO, sweat gland output; ISO, isosmotic condition; HYP, hyperosmotic condition.

* Significantly different from females (*P* ≤ 0.05). Values represent mean ± SD.

#### Cutaneous vascular conductance

**Figure 3.**
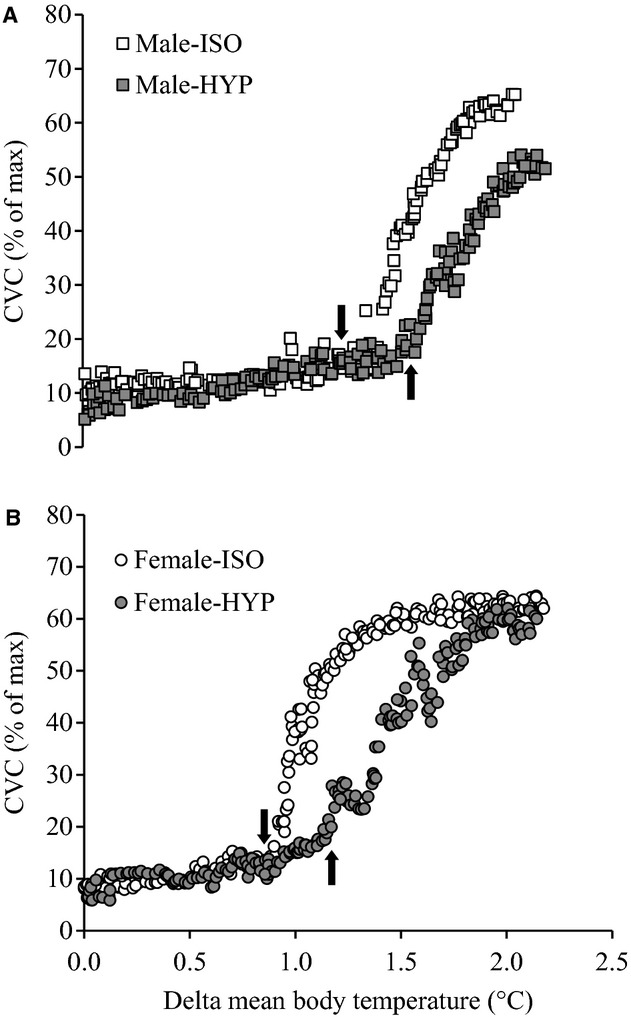
Forearm cutaneous vascular conductance (CVC) response as a function of increases in mean body temperature during whole‐body passive heating in isotonic (open symbols) and hypertonic (closed symbols) conditions for two representative subjects (A: male; B: female). Arrows indicate the onset threshold for CVC.

Table 3 shows the mean body temperature absolute onset threshold, change in onset threshold, and thermosensitivity of CVC in both males and females. Absolute mean body temperature onset threshold of CVC was significantly higher during HYP when compared to ISO (*P* = 0.001). In addition, a main effect of sex was observed in the absolute threshold of CVC (*P* = 0.003) with females having a higher absolute threshold. The change in mean body temperature from baseline to the onset of CVC was significantly higher during HYP (*P* < 0.001), however, no differences were observed between males and females (*P* = 0.275). Finally, no differences the thermosensitivity of CVC were observed between conditions (ISO vs. HYP, *P* = 0.621) or sexes (males vs. females, *P* = 0.601). The CVC response of two representative participants during ISO and HYP conditions is shown in Figure 3.

### Cardiovascular responses

**Figure 4.**
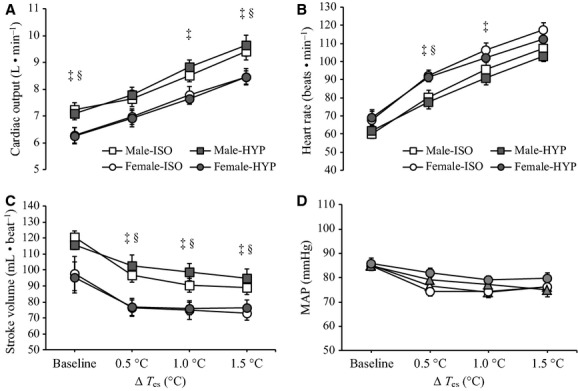
Mean cardiac output (A), heart rate (B), stroke volume (C), and mean arterial pressure (MAP) (D) for both males (□) and females (○) during isosmotic (ISO) and hyperosmotic (HYP) conditions at baseline and throughout whole‐body passive heating. Values represent mean ± standard error. ^‡^Male‐ISO is significantly different from Female‐ISO and §Male‐HYP is significantly different from Female‐HYP (*P* ≤ 0.05).

Table 5 outlines baseline values of cardiac output, heart rate, stroke volume, and MAP for both males and females during ISO and HYP conditions. Baseline heart rate and stroke volume did not differ across conditions (*P* = 0.107 and *P* = 0.132) or sexes (*P* = 0.173 and *P* = 0.059). Cardiac output at baseline was consistently lower in females during both ISO (*P* = 0.036) and HYP (*P* = 0.027) conditions. However, no main effect of condition was observed (*P* = 0.735). During whole‐body heating, heart rate and cardiac output significantly increased (all *P* < 0.001), while stroke volume significantly decreased (*P* < 0.001). No main effect of condition was observed for the changes in either response with increases in esophageal temperature (all *P* > 0.1). Changes in heart rate, cardiac output, and stroke volume as a function of increases in esophageal temperature are shown in Figure 4. No differences in MAP were observed between males and females (*P* = 0.630); however, MAP significantly decreased (*P* < 0.001) during passive heating, and to the same extent in both ISO and HYP conditions (*P* = 0.085).

**Table 5.** Baseline values for cardiac output, heart rate, stroke volume, and MAP during ISO and HYP conditions.

Condition	Sex	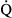 (L/min)	HR (bpm)	SV (mL)	MAP (mmHg)
ISO	Male	7.2 ± 0.8^*^	60 ± 8	120.8 ± 11.2	85 ± 3
	Female	6.3 ± 0.9	68 ± 14	97.7 ± 32.1	85 ± 6
HYP	Male	7.1 ± 0.7^*^	62 ± 9	116.0 ± 10.0	85 ± 3
	Female	6.3 ± 0.9	69 ± 14	95.3 ± 29.2	86 ± 6

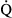
, cardiac output; HR, heart rate; SV, stroke volume; MAP, mean arterial pressure; ISO, isosmotic condition; HYP, hyperosmotic condition.

*Significantly different from females (*P* ≤ 0.05). Values represent mean ± standard deviation.

## Discussion

The purpose of this study was to examine if there are sex‐related differences in the osmotically induced changes in both sweating and CVC. The main finding is that the overall delay in onset threshold of sweating and CVC was similar between males and females. We hypothesized that an increase in plasma osmolality would result in a delayed onset threshold of sweating and CVC in both sexes. Congruent with this hypothesis, our results showed a delayed onset threshold in both thermoefferent responses in males and females. However, our results did not support our second hypothesis which stated that the overall delay in onset threshold would be greater in females than males. On the contrary, there were no differences in the overall change in mean body temperature required to elicit an increase in sweating and CVC between sexes.

### Sweating

This study showed a higher onset threshold of sweating during the HYP condition in both males and females. This delay in onset threshold of local sweating after inducing a HYP state supports previous findings by others (Fortney et al. 1984; Takamata et al. 1997; Lynn et al. 2012) and further expands on these studies by showing, for the first time, the effects of hyperosmolality in young females. Furthermore, our results showed that while hyperosmolality resulted in a delayed onset threshold of sweating, there were no differences in the overall delay between males and females. To the best of our knowledge, no studies to date have conducted a direct comparison between sexes on the effects of hyperosmolality on the local sudomotor response. Nonetheless, similar to our results in the ISO condition, studies examining sex‐related differences in heat loss responses have reported no differences in the onset threshold of sweating between males and females during passive heat stress (Inoue et al. 2005; Gagnon et al. 2013). The present study also showed that sweating thermosensitivity was consistently lower in females irrespective of osmotic state (ISO vs. hypertonic). While a lower sweating thermosensitivity in females has been previously reported during passive heat stress (Gagnon et al. 2013) and exercise (Gagnon and Kenny 2012b), our results further expand on previous observations on females by showing that the reduced sweating thermosensitivity is also evident in a HYP state. More importantly, our findings suggest that the extent to which sweating thermosensitivity is reduced in females is not affected by hyperosmolality, as the slope of the response did not differ between osmotic conditions. Until recently, the idea that hyperosmolality does not affect sweating thermosensitivity had only been shown in males (Takamata et al. 1997; Lynn et al. 2012), however, our results demonstrate that previous observations in males also extend to females.

Measurements of heat‐activated sweat glands in the forearm at the end of heating showed no differences between males and females. Therefore, the reduced sweating response observed in females is mainly driven by lower sweat gland output. In keeping with our results, previous studies have reported no differences in heat‐activated glands in the forearm, while having lower sweat gland outputs in females during both passive heating (Inoue et al. 2005; Gagnon et al. 2013) and exercise (Gagnon and Kenny 2012b). However, no studies to date examining the effects of hyperosmolality on heat loss responses have assessed sweat gland activation in either males or females. Therefore, this study is the first to show that after controlling for osmotic state, there were no sex‐related differences in sweat gland activation and sweat gland output between conditions (isotonic vs. hypertonic). This suggests that hyperosmolality does not affect either one of these variables. These observations expand the current literature on the effects of hyperosmolality on sudomotor response in males, and provide novel information on the female response. It should be noted that higher sweat gland activation in females has been reported in the chest and upper back during exercise (Gagnon and Kenny 2012b), which suggests regional variations in sweat gland activation. This regional variation cannot be assessed from the present study given that local sweating and sweat gland activation was only measured in the forearm. Future research should evaluate the potential influence of hyperosmolality on regional variation in sudomotor response in both males and females.

Our results demonstrate that (1) there are no sex‐related differences in the onset threshold of sweating, (2) the overall delay in the onset threshold of sweating was similar between sexes, and (3) females exhibited consistently lower sweating thermosensitivity than males irrespective of plasma osmolality. Taken together, these findings suggest that previously reported differences in sudomotor activity between males and females are not modulated by changes in blood osmolality via osmoreceptors. To the best of our knowledge, the study by Gagnon and Kenny (2012b) has been the only study to report plasma osmolality when examining sex‐related differences in thermoeffector activity (sweating and skin blood flow) during incremental exercise. However, plasma osmolality was only measured in a subgroup of participants during a separate experimental session and heat loss responses were not reported in this subgroup. Therefore, the relationship between plasma osmolality and thermoefferent responses could not be established. Nonetheless, the lack of differences in plasma osmolality in this subgroup of males and females led the authors to speculate that osmoreceptors were most likely not involved in the reduced sudomotor response observed in females. In fact, the present study provides, for the first time, direct evidence that reduced sweat rate in females cannot be attributed to hyperosmolality, and therefore, these sex‐differences in sudomotor response are likely mediated by an alternative mechanism. As previously suggested by Inoue et al. (2005) these differences may therefore be at the end‐organ level (i.e., sweat gland) as either differences in the morphology or cholinergic sensitivity of the sweat gland. However, the recent study by Gagnon et al. (2013) discarded the latter possibility by showing no differences in the response to cholinergic agonists between males and females. As concluded by Gagnon et al. (2013) sex‐related differences in sweating may therefore be in the morphology of the gland (i.e., size of the sweat gland) and future research should examine this possibility.

### Cutaneous vascular conductance

This study showed that hyperosmolality results in a delayed onset threshold of CVC, which supports findings by previous studies (Fortney et al. 1984; Takamata et al. 1997; Shibasaki et al. 2009; Lynn et al. 2012). This study also showed that the onset threshold of CVC is not different between males and females in either condition (iosmotic vs. HYP). Even though no previous studies have compared the effects of hyperosmolality on the onset threshold of CVC between males and females, some recent studies examining sex‐related differences in CVC during passive heating reported no differences in onset thresholds between sexes (Inoue et al. 2005; Gagnon et al. 2013). While this matches our findings during the ISO condition, this study is the first to report such changes during a HYP state. These observations suggest that the overall delay in the onset threshold induced by hyperosmolality occurs to the same extent in both males and females. Furthermore, our results showed no differences in the thermosensitivity of CVC between males and females, which is in keeping with recent studies using passive heat stress (Inoue et al. 2005; Gagnon et al. 2013). Finally, the thermosensitivity of CVC did not differ with osmotic conditions, which is similar to previous results on males during passive heating (Takamata et al. 1997; Lynn et al. 2012). However, this is the first study to show such results in females.

### Limitations

This study was designed to examine osmoreceptor modulation only; as such, baroreceptor activity was not assessed. This is an important limitation given that baroreceptor unloading alone can result in a delayed onset threshold of CVC (Crandall et al. 1996; Cui et al. 2004; Lynn et al. 2012) and when combined with hyperosmolality it can have an additive effect (Lynn et al. 2012). Nonetheless, studies examining the effects of baroreceptor unloading on CVC, using consecutive increments of lower body negative pressure, have shown that the onset threshold of CVC was only affected at higher levels of lower body negative pressure, usually greater than −21 mmHg (Crandall et al. 1996; Cui et al. 2004). Taking this into consideration, along with the fact that this study was conducted in the supine posture and no differences in MAP were observed between groups and conditions, it is unlikely that baroreceptor unloading may have confounded our results. However, if baroreceptor unloading played a role in our results, it would have been to the same extent in both sexes given that no differences in MAP were observed between groups. Furthermore, it should be noted that while there is still significant disagreement on the effects of baroreceptor unloading on sudomotor response (Shibasaki et al. 2006), a recent study by Lynn et al. (2012) demonstrated that unloading the baroreceptors had no effect in local sweating during passive heating in an ISO and HYP condition. Therefore, we are confident that our sweating responses are not attributed to baroreceptor loading status. Given that our experimental design does not allow us to detect the influence, if any, of baroreceptors further research should examine sex‐related differences and the combined effect of baroreceptors and osmoreceptors in local heat loss responses.

Female participants were asked to complete both experimental sessions during the follicular/low hormone phase of the menstrual cycle, therefore, our results cannot be extended to other phases. Further research is needed to evaluate the effects of different phases of the menstrual cycle on the HYP inhibition of thermoeffector responses (sweating and CVC). Finally, our sample consisted of young men and women, therefore, our results are limited to this specific population, and cannot be extrapolated to older adults or populations with chronic conditions such as diabetes or obesity. The sex‐related differences in osmoreceptors modulation of local heat loss responses on these vulnerable populations remain to be elucidated.

### Perspectives

This study provides novel information on the effects of hyperosmolality on thermoefferent responses in females. This work showed that while the onset threshold of both sweating and CVC increased in the HYP condition, this delay was similar between males and females. Furthermore, females also exhibited a reduced sweating thermosensitivity and sweat gland output irrespective of condition. Although the lack of differences between males and females on the delay in onset thresholds may point toward a similar response to a given level of osmolality, the net effect on the capacity to dissipate heat may be very different. It is well‐accepted that changes to the onset threshold or the thermosensitivity of thermoeffector responses can profoundly affect heat dissipation, and therefore, heat storage. For instance, a delay in onset threshold and reduction in thermosensitivity will compromise the ability to achieve heat balance, resulting in greater heat storage (Gagnon and Kenny 2012a). This is particularly true for females since, as shown by the present study, they display a delay in onset threshold compounded by an already reduced sweating thermosensitivity. As a result, females will store more heat before achieving heat balance.

## Conclusion

In summary, this study examined if there are sex‐related differences in the osmotically induced changes in both sweating and CVC. Our results suggest that the osmotically induced delay in the onset threshold of sweating and CVC is similar between males and females. Although the thermosensitivity of CVC was similar between sexes, the thermosensitivity of sweating was consistently lower in females compared to males. The lower thermosensitivity in sudomotor response of females was accompanied by a lower sweat gland output, albeit similar sweat gland activation to males. As a result, the sex‐related differences in thermoeffector activity are independent of osmoreceptor activation. We conclude that osmoreceptors do not exhibit a sex‐related dependent modulation of sweating and CVC responses during heat stress, and therefore, the lower sudomotor activity of females cannot be attributed to osmoreceptor modulation.

## Acknowledgments

The authors would like to thank Daniel Gagnon for his invaluable contributions to the study. Also, we would like to specially thank all the participants who volunteered their time to participate in this study.

## Conflict of Interest

None declared.
